# Deep sustained response to daratumumab monotherapy associated with T-cell expansion in triple refractory myeloma

**DOI:** 10.1186/s40164-018-0096-7

**Published:** 2018-02-07

**Authors:** Saad Z. Usmani, Imran Khan, Christopher Chiu, David Foureau, Lawrence J. Druhan, Katherine Rigby, Tineke Casneuf, A. Kate Sasser

**Affiliations:** 1 Levine Cancer Institute/Carolinas Health Care System, 1021 Morehead Medical Drive, Charlotte, NC 28204 USA; 2 Janssen Research & Development, LLC, Raritan, NJ USA; 3 Janssen Research & Development, LLC, Spring House, PA USA; 4 Janssen Research & Development, Beerse, Belgium

**Keywords:** Daratumumab, Immune response, Multiple myeloma, Stringent complete response, Sustained response, T-cell expansion, Triple refractory, Minimal residual disease

## Abstract

**Background:**

Daratumumab, a human CD38 monoclonal antibody that has direct on-tumor and immunomodulatory mechanisms of action, demonstrated clinical benefit as monotherapy or in combination with established regimens in patients with multiple myeloma with one or more prior lines of therapy.

**Case presentation:**

A male patient, who was 70 years of age at the time of diagnosis of multiple myeloma in 2011, relapsed after five lines of therapy, including autologous stem cell transplantation. The patient’s disease, which was considered high risk with a deletion of chromosome 17p, advanced quickly and was triple refractory 2 years after diagnosis leaving few treatment options. He was treated with daratumumab monotherapy in the SIRIUS clinical trial resulting in a stringent complete response and clearance of minimal residual disease. The duration of the patient’s clinical response is now over 3.5 years without relapse, compared with a median of 7.6 months for similarly treated patients. The patient’s immunophenotype revealed CD8^+^ T-cell expansion, clonal expansion of the T-cell receptor repertoire, and decreases in regulatory T cells during daratumumab therapy, suggesting a robust adaptive immune response. This immune response was still present 32 months into daratumumab therapy.

**Conclusions:**

The results from this case report showed that a patient with advanced multiple myeloma, who had exhausted all treatment options with existing regimens, mounted an ongoing, deep, and durable response to daratumumab monotherapy. Further investigation of the immunologic profile provided additional patient-level evidence of an immunomodulatory mechanism of action of daratumumab.

*Trial registration* ClinicalTrials.gov Identifier number NCT01985126. Submitted 22 July 2013

**Electronic supplementary material:**

The online version of this article (10.1186/s40164-018-0096-7) contains supplementary material, which is available to authorized users.

## Background

Daratumumab, a human monoclonal antibody targeting CD38, has shown clinical benefit as monotherapy or in combination with established regimens in patients with multiple myeloma treated with at least one prior therapy [1–4]. Outcomes are poor in myeloma patients relapsed from or refractory to multiple lines of treatment. A recent real-world assessment of outcomes in patients who had received at least three prior lines of therapy, including an alkylating agent, and were refractory to both an immunomodulatory drug and a proteasome inhibitor showed a median overall survival of 13 months [5]. In the SIRIUS study of daratumumab monotherapy (16 mg/kg) in a similarly heavily treated refractory patient population, an overall response rate of 29%, including three stringent complete responses (sCRs), was accompanied by median overall survival of 18.6 months [3, 6].

The antimyeloma activity of daratumumab is mediated through distinct on-tumor mechanisms, including antibody-dependent cellular cytotoxicity, complement-dependent cytotoxicity, macrophage-mediated phagocytosis, and apoptosis via Fc-mediated crosslinking [7–9]. In myeloma patients, numbers of CD38^+^ regulatory T cells, CD38^+^ regulatory B cells, and CD38^+^ myeloid-derived suppressor cells are depleted by daratumumab treatment [10]. As a result of this activity numbers of CD4^+^ T-helper cells and CD8^+^ cytotoxic T cells T cell numbers rise, along with an increase in clonality, providing a prolonged and ongoing additional immunomodulatory mechanism of action [10]. In this report, we describe a deep clinical response of over 3.5 years, including eradication of minimal residual disease (MRD), in a heavily treated, deletion 17p myeloma patient who was enrolled in the SIRIUS study. This clinical response was associated with robust immune profile changes including CD8^+^ T-cell expansion, increased clonality, and decreased regulatory T cells while on daratumumab treatment.

## Case presentation

In September 2010 at the Levine Cancer Center (North Carolina, USA), a 70-year-old male presented with a plasmacytoma of the right eleventh rib. A bone marrow biopsy showed 10% clonal plasma cells, negative 24-h urine protein electrophoresis, a serum protein level of 0.3 g/dL (IgA kappa), and a kappa-to-lambda ratio of 1.96. The patient received local external radiotherapy in December 2010, and no active lesions were detected by positron emission tomography–computed tomography in January of the following year. In October 2011, the patient was diagnosed with multiple myeloma (IgA kappa) of stage 1 by the International Staging System [11] and stage 3 by the Durie Salmon Staging System [12]. Analysis of plasma cells by fluorescence in situ hybridization showed that 7.5% carried a chromosome 17p deletion, a marker of high-risk disease. The patient also had lytic bone lesions involving the axial and appendicular skeleton with osteopenia. The patient received induction therapy of one cycle of lenalidomide and dexamethasone (minimal response) and five cycles of bortezomib, lenalidomide, and dexamethasone (partial response) between December 2011 and June 2012, prior to an autologous stem cell transplantation in September 2012. The patient achieved a very good partial response and remained on maintenance therapy of bortezomib weekly (1.3 mg/m^2^) and 10 mg lenalidomide daily on Days 1–21 of each 4-week cycle until disease progression in March 2013. In April 2013, the patient received pomalidomide 4 mg orally on Days 1–21 of each 4-week cycle, bortezomib weekly (1.5 mg/m^2^), and dexamethasone and achieved a partial response prior to disease progression after 6 cycles.

In October 2013, having become triple refractory during the 2 years following diagnosis, the patient was enrolled in the SIRIUS study and received daratumumab monotherapy at the approved dosing schedule [6]. The patient provided written informed consent to participate in the clinical trial and additional consent for the analyses in this report. The study design and results from the SIRIUS study have been reported in detail elsewhere [6].

**Fig. 1:**
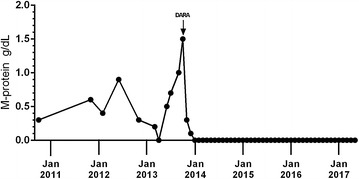
M-protein levels over time. After initial presentation in 2010, the patient received five rounds of treatment and relapsed each time. The patient received the first dose of daratumumab (16 mg/kg) in October 2013 when he enrolled in the SIRIUS study. M-protein levels were measured at the local laboratory and then centrally after the first dose of daratumumab

There were no reported infusion-related reactions during the first or subsequent infusions for this patient. He achieved a partial response after 28 days as assessed by a computerized algorithm based on the International Myeloma Working Group (IMWG) response criteria [13]. A very good partial response was reported after 56 days, and the patient achieved an sCR, marked by no detectable disease by IMWG criteria, in May 2014 (194 days after the first dose). The patient has maintained this sCR for over 3.5 years; as of May 2017 while receiving daratumumab 16 mg/kg once every 4 weeks. The patient’s M-protein levels over time and the response to treatment are shown in Fig. 1 [6].

The patient’s baseline immune profile was assessed prior to his first dose of daratumumab in the SIRIUS study. Blood and bone marrow samples were stained with multi-fluorochrome antibody panels and a FITCα-CD38 (003HuMax; Genmab A/S, Copenhagen, Denmark and Janssen Research & Development, Spring House, Pennsylvania, USA), an antibody that binds to an epitope distinct from the epitope bound by daratumumab. Blood samples were lysed with FACS™ lysing solution (BD, San Jose, California, USA), and FIX and PERM^®^ cell permeabilization reagent (Invitrogen, Waltham, Massachusetts, USA) was used for bone marrow aspirates. Analysis was performed using FACS Canto II flow cytometers, and data were analyzed using FACSDiva™ software (BD). Absolute cell counts were calculated for peripheral blood samples, and immune cell subpopulations were represented as a percentage of total lymphocytes for bone marrow samples. The data cutoff was December 31, 2015.

**Fig. 2:**
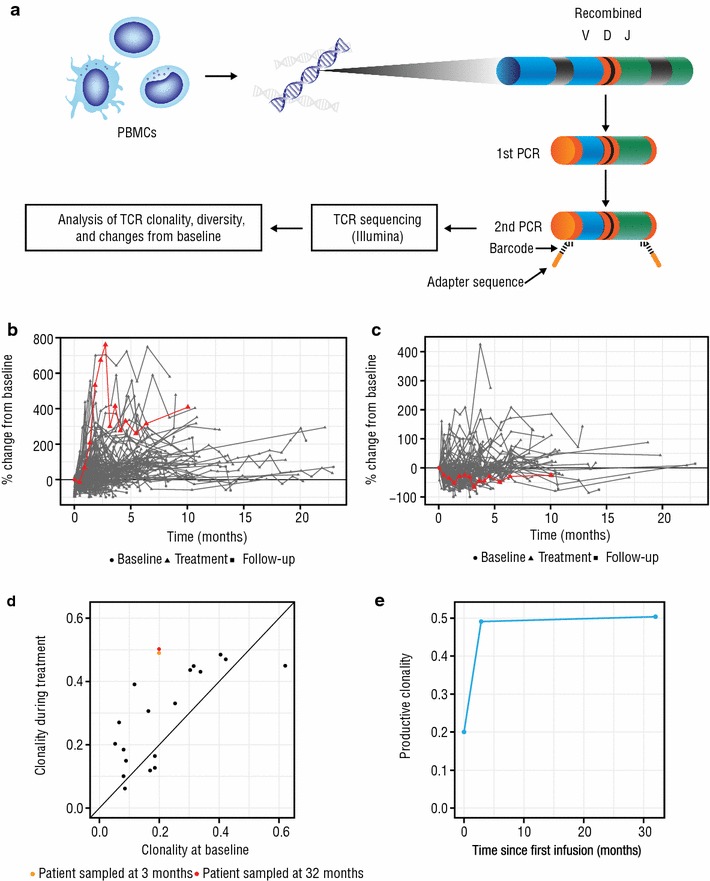
Change in immune cell populations and properties. A diagram of the T-cell receptor sequencing protocol is shown in **a**. DNA was extracted from peripheral blood mononuclear cells and subjected to 2 rounds of PCR amplification. During the first round, primers specific for the variable (forward) and joining (reverse) were used to amplify somatically recombined T-cell receptors. During the second round, each amplicon was amplified using primers containing barcode and adapter sequences to facilitate T-cell receptor sequencing via the Illumina platform in the next step of the protocol. The sequencing data were used to evaluate T-cell receptor clonality, diversity, and changes from baseline. Percent changes in CD8 T cells from baseline among patients in the SIRIUS study are shown in **b**. The patient, indicated by the red line, showed a rapid expansion of CD8^+^ T cells that was maintained over time. **c** The percent change from baseline of regulatory T cells over time. **d** T-cell receptor clonality at baseline versus on-treatment time points. For all patients in black, the on-treatment time point was 3 months. For the patient, the sample time points were 3 and 32 months, in orange and red, respectively. **e** The T-cell receptor clonality in the case study patient at baseline, 3, and 32 months. *PBMCs* peripheral blood mononuclear cells, *V* variable region, *D* diversity region, *J* joining region, *PCR* polymerase chain reaction, *TCR* T-cell receptor

DNA from frozen patient peripheral blood mononuclear cells was evaluated for T-cell receptor sequences with the ImmunoSEQ™ assay (Adaptive Biotechnologies, Seattle, Washington, USA) [14] (Fig. 2a). The assay used prequalified multiplex polymerase chain reaction (PCR) assays (TR2015CRO-V-019) composed of forward and reverse primers that directly targeted the family of variable (V) genes (forward primers) and joining (J) genes (reverse primers). Each V and J gene primer served as priming pairs to amplify somatically recombined T-cell receptors, with each primer having a specific universal DNA sequence. After initial PCR amplification, each amplicon was then amplified again with forward and reverse primers containing the universal sequence and adaptor sequence necessary for DNA sequencing by Illumina (San Diego, California, USA). T-cell receptor sequences were analyzed to determine clonality, diversity, and changes from baseline. The extent of mono- or oligoclonal expansion was calculated and quantitated by measuring the shape of the clone frequency distribution. Values ranged from 0 to 1, where values approaching 1 indicated a nearly monoclonal population:

$$ Entropy = H = {-}\mathop \sum \limits_{i - 1}^{N} p_{i} { \log }_{2} (p_{i} ) $$

and

$$ Clonality = 1 - \frac{H}{{{ \log }_{2} (N)}} $$

where N was the total number of unique T-cell receptor β clones and p_i_ was the frequency of the ith unique T-cell receptor β clone [15].

Immune correlatives were analyzed in June 2016. The patient’s baseline peripheral levels of natural killer, B, and T cells (CD3, CD4, CD8) were similar to the levels of other patients enrolled in the study (median [and standard deviation] of all patients and this patient, respectively: CD3: 614 [428.4] and 509 × 10^6^/L, CD4: 233 [173.1] and 355 × 10^6^/L, CD8: 317 [315.5] and 157 × 10^6^/L). However, he had elevated baseline levels of regulatory T cells compared with other patients (median [and variance] of all and this patient, respectively: 23 [14.24] and 51 × 10^6^/L. In the SIRIUS study, most daratumumab-treated patients experienced T-cell expansion that was driven primarily by CD8^+^ T cells. The expansion of CD8^+^ T cells in this patient was among the largest in the study population, resulting in an increase of approximately 800% from baseline by 3 months following the first daratumumab dose (Fig. 2b). The expansion of the CD8^+^ T-cell population in this patient was accompanied by a decrease in regulatory T cells of 67% at 3 months (Fig. 2c).

The T-cell repertoire is an informative biomarker for assessing a patient’s immune status and response to immune modulation. We had previously shown by PCR next-generation sequencing of the T-cell repertoire that T-cell expansion was clonal in patients treated with daratumumab monotherapy [10]. Additionally, patients with a clinical response to daratumumab had significantly greater increases in both expansion of individual clones and in the sum of all expanded clones. Changes in T-cell receptor clonality from baseline to 3 months of daratumumab treatment in 16 different patients enrolled in the SIRIUS study are shown in Fig. 2d. The patient had the greatest change in clonal T cells from baseline after 3 months. This clonal T-cell expansion was sustained for 32 months (Fig. 2e). During this period, the patient maintained an sCR and continues on therapy today.

Other factors have been associated with clinical response to anti-myeloma and daratumumab therapy. The percentages of bone marrow plasma cells at baseline, or complement proteins (C1q, C2, C3, and C4), were not significantly different in this patient compared with other study participants. Reductions in natural killer cells and the profiles of B cells and monocytes during daratumumab treatment were all similar in the patient compared with other SIRIUS study participants. Baseline levels of CD38 and the complement inhibitory proteins CD55 and CD59 were not measured in this patient [16].

**Fig. 3:**
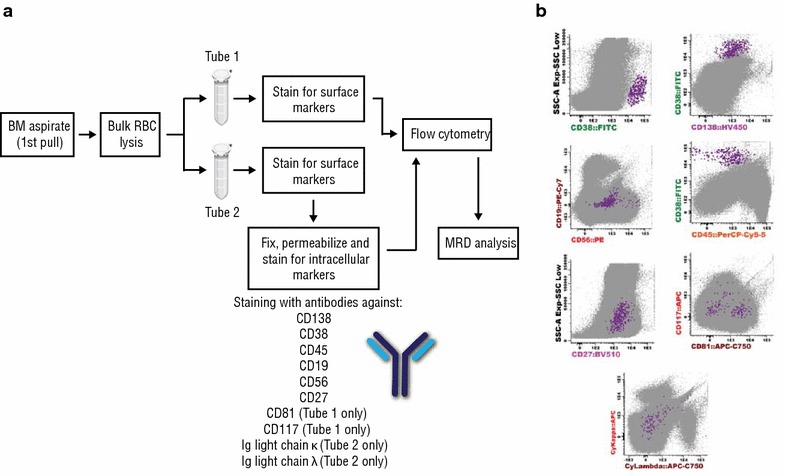
Minimal residual disease. A diagram of the protocol used to assess minimal residual disease shown in **a**. Bone marrow aspirates subjected to bulk red blood cell lysis and separated into 2 tubes. Each tube was incubated with an 8-marker antibody combination. Tube 1 was stained for surface markers only and tube 2 surface and intracellular markers. Minimal residual disease was then measured by flow cytometry merging data from each tube into one analysis. As shown in **b** plasma cells (PC) displayed a CD38^high^ CD138^int^ phenotype with low CD19 and CD45 expression indicative of an immature plasmablast (PC) population. By applying a cutoff value of 1 abnormal/clonal plasma cell per million nucleated event for MRD positivity (i.e., 10^−6^ sensitivity threshold of MRD positivity), the bone marrow aspirate tested MRD negative. *BM* bone marrow, *RBCs* red blood cells, *MRD* minimal residual disease

The patient was also assessed for MRD by flow cytometry in December 2015. Flow cytometry for MRD detection was based on an assay developed by the EuroFlow Consortium [15]. Briefly, bone marrow aspirates were incubated post red cell lysis (Pharm Lyse™ Buffer, BD Biosciences, San Jose, California, USA) in two separate tubes containing 10-marker antibody combinations against CD138, CD38, CD45, CD19, CD56, CD81, CD117, CD27, and immunoglobulin κ and/or λ (see Additional file 1: Table S1) (Fig. 3a). Red cells in both tubes (each containing 6 million cells) were lysed and stained for surface markers (30 min, room temperature). The second tube was also fixed and permeabilized (FIX & PERM ™ Cell Permeabilization Kit by Thermo Fisher Scientific, Rochester NY, USA) for intracellular staining (15 min, room temperature. Five million events from each tube were acquired using a 14-color BD FACS Aria II flow cytometer (configured as described in Additional file 1: Table S2) for a total of 10 million cells assessed for the analysis. Flow cytometry standard files for both tubes were combined as a single data file for the analysis using Infinicyt™ flow cytometry software version 1.8 (Cytognos S.L., Salamanca, Spain). The patient was MRD negative at the 10^−5^ sensitivity threshold (one cancer cell in 100,000 normal cells; Fig. 3b). The plasma cell distribution represented 0.0035% of total nucleated cells, with no phenotypic abnormalities or kappa-to-lambda ratio bias.

## Discussion and conclusions

This patient had previously received many lines of antimyeloma treatment within a short time frame, and his prognosis was poor. Upon receiving daratumumab monotherapy, the patient responded to treatment within 1 month and continued to improve over time to a point where MRD (at a 10^−5^ sensitivity threshold) was not detectable. Maintenance of this response for over 3.5 years was associated with the recently described immunomodulatory effects of daratumumab [10], including CD8^+^ T-cell expansion and increased clonality, along with decreased regulatory immune cell populations. In the SIRIUS study, 29% of patients responded to treatment with only three sCRs, the longest of which was observed in this patient. However, in a pooled analysis of SIRIUS and GEN501 [17], another early-phase study of daratumumab, patients with a best outcome of stable disease or minimal response also showed prolonged median survival of 18.5 months [18]. It was hypothesized that exposure to daratumumab may have enabled an enhanced response to the next therapy. As the patient population in the SIRIUS study was very heavily treated, it is of interest whether daratumumab may have a greater effect in patients with less-compromised immune systems and also in combination with other antimyeloma agents. The CASTOR study of daratumumab plus bortezomib and dexamethasone in patients with a median of two lines of therapy showed an overall response rate of 82.9% versus 63.2% in the control arm [1]. In the POLLUX study of daratumumab plus lenalidomide and dexamethasone in patients with a median of one prior line of therapy, the overall response rate was 92.9% versus 76.4% after a longer median follow-up compared with CASTOR [2]. A preliminary analysis of POLLUX showed that robust increases in T-cell clonality were observed in the daratumumab arm but not in the control group [19].

The results from this case report expand upon data supporting an important immunomodulatory mechanism of action of daratumumab and suggest that aspects of the antimyeloma activities of daratumumab may be further enhanced by immuno-oncology combinations of programmed cell death-1 or programmed death ligand-1 inhibitors in multiple myeloma.

## Additional file

**Additional file 1: Table S1.** Antibodies used for flow cytometry–based minimal residual disease assay and immune phenotyping. **Table S2.** Configuration of BD FACSAria II for minimal residual disease analysis.

## Abbreviations

IMWG: International Myeloma Working Group
J: joining
MRD: minimal residual disease
PCR: polymerase chain reaction
sCR: stringent complete responses
V: variable
