# Supplementary material for: Deep sustained response to daratumumab monotherapy associated with T-cell expansion in triple refractory myeloma
Source: Exp Hematol Oncol. 2018 Feb 7;7:3. doi: 10.1186/s40164-018-0096-7 (PMC5804097; doi:10.1186/s40164-018-0096-7)
Supplement: Supplementary file 1 — Additional file 1: Table S1. Antibodies used for flow cytometry–based minimal residual disease assay and immune phenotyping. Table S2. Configuration of BD FACSAria II for minimal residual disease analysis. [file 40164_2018_96_MOESM1_ESM.docx]

**SUPPLEMENTARY TABLES**

**Table of Contents**

**Page**

Table S1. Antibodies used for flow cytometry–based minimal residual disease 3

assay and immune phenotyping

Table S2. Configuration of BD FACSAria II for minimal residual disease analysis 4

**Table S1. Antibodies used for flow cytometry–based minimal residual disease assay and immune phenotyping**

| **Marker** | **Channel** | **Clone** | **Supplier** |
| --- | --- | --- | --- |
| **MM MRD Assay** |  |  |  |
| CD38 | FITC | Multiepitope | ALPCO |
| CD56 | PE | C5.9 | ALPCO |
| CD45 | PerCP-Cy5.5 | HI30 | BioLegend |
| CD19 | PE-Cy7 | J3-119 | ALPCO |
| CD117 | APC | 104D2 | BD Biosciences |
| CD81 | APC-C750 | M38 | ALPCO |
| CD138 | HV450 | MI 15 | BD Biosciences |
| CD27 | BV510 | O323 | BioLegend |
| Anti-Kappa | APC | Polyclonal | Dako |
| Anti-Lambda | APC-C750 | Polyclonal | ALPCO |

**Table S2. Configuration of BD FACSAria II for minimal residual disease analysis.**

| **Laser** | **Excitation (nm)** | **Detector** | **Dichroic Mirror** | **Bandpass Filter** |
| --- | --- | --- | --- | --- |
| Blue Argon Laser | 488 | FSC |  |  |
|  |  | SSC |  | 488/10 |
|  |  | FITC | 502LP | 530/30 |
|  |  | PE | 556LP | 585/42 |
|  |  | PE-CF594 | 610LP | 616/23 |
|  |  | PerCP-Cy5.5 | 695LP | 695/40 |
|  |  | PE-Cy7 | 735LP | 780/60 |
| Red Helium-neon Laser | 633 | Alexa647 or APC |  | 660/20 |
|  |  | APC-C750 | 750LP | 780/60 |
| Violet Diode | 405 | BV421 or HV450 |  | 450/50 |
|  |  | BV510 | 475LP | 525/50 |

Stream-in-air square flow cell type, sheath pressure 20 psi, nozzle size 100 μm. Diva version 8 software for data acquisition.
